# Parametrization of the Calcaneus and Medial Cuneiform to Aid Potential Advancements in Flatfoot Surgery

**DOI:** 10.3390/life14030328

**Published:** 2024-02-29

**Authors:** Yanni Cai, Giulia Pascoletti, Peter Zioupos, Basil Budair, Elisabetta M. Zanetti, Trevor J. Ringrose, Sarah Junaid

**Affiliations:** 1 Cranfield Forensic Institute, Defence Academy of the United Kingdom, Cranfield University, Swindon SN6 8LA, UK; yanni.cai@cranfield.ac.uk (Y.C.);; 2 School of Mechanical, Biomedical and Design Engineering, Aston University, Birmingham B4 7ET, UK; 3 Department of Engineering, University of Perugia, 06125 Perugia, Italy; giulia.pascoletti@unipg.it (G.P.);; 4 Biomedical Engineeering, School of Engineering, University of Hull, Kingston-upon-Hull HU6 7RX, UK; p.zioupos@hull.ac.uk; 5 Royal Orthopaedic Hospital (ROH), Birmingham B31 2AP, UK

**Keywords:** pes planus, flatfoot, principal component analysis, parametrization, calcaneus, medial cuneiform

## Abstract

Introduction: Flatfoot is a condition commonly seen in children; however, there is general disagreement over its incidence, characterization and correction. Painful flatfoot accompanied with musculoskeletal and soft tissue problems requires surgery to avoid arthritis in adulthood, the most common surgical approach being two osteotomies to the calcaneus and medial cuneiform bones of the foot. Objectives: This study focuses on the parametrization of these two bones to understand their bone morphology differences in a population sample among 23 normal subjects. Population differences could help in understanding whether bone shape may be an important factor in aiding surgical planning and outcomes. Methods: A total of 45 sets of CT scans of these subjects were used to generate surface meshes of the two bones and converted to be iso-topological meshes, simplifying the application of Generalized Procrustes Analysis and Principal Component Analysis, allowing the main sources of variation between the subjects to be quantified. Results: For the calcaneus, 16 Principal Components (PCs) and, for the medial cuneiform, 12 PCs were sufficient to describe 90% of the dataset variability. The quantitative and qualitative analyses confirm that for the calcaneus PC1 describes the Achilles attachment location and PC2 largely describes the anterior part of the bone. For the medial cuneiform, PC1 describes the medial part of the bone, while PC2 mainly describes the superior part. Conclusion: Most importantly, the PCs did not seem to describe the osteotomy sites for both bones, suggesting low population variability at the bone cutting points. Further studies are needed to evaluate how shape variability impacts surgical outcomes. Future implications could include better surgical planning and may pave the way for complex robotic surgeries to become a reality.

## 1. Introduction

Pes planus of the foot, an idiopathic [1] condition commonly seen in children which may continue to adulthood, is associated with a low or absent medial longitudinal arch and some degree of hindfoot and forefoot malalignment [2]. The true incidence of flatfoot is unknown; however, many research studies have tried to understand this condition [3,4,5,6,7,8], with conflicting outcomes contributing to a general disagreement even on its explicit definition and the differences between a “normal” foot and a flatfoot [9]. For this reason, flatfoot is often considered as a normal variant of the foot shape rather than a condition [9]. There are mainly three types of flatfeet [10]: the flexible flatfoot (FFF), which is usually asymptomatic and most common, the flexible flatfoot with short Achilles tendon (FFF-STA), which affects one in four people with FFF, causing pain and often requiring surgery, and the rigid flatfoot, which is always symptomatic and caused by neuromuscular disorders [11]. The term “flexible” means that the foot appears flat when it is weight-bearing (when the patient stands up), while it returns to normal when the foot is no longer weight-bearing (when the patient sits down with their feet suspended) [12].

Corrective surgery in FFF is recommended in adolescents only for symptomatic cases to prevent disability and arthritis developing in adulthood [13] when other conservative methods such as orthotics have failed to relieve pain [9,14]. However, surgery brings its own challenges. Given the complex foot structure and the not-fully understood foot biomechanics, there is no universal agreement on how to treat flatfoot successfully, and no optimal surgical methodology has yet been established [15]. Surgical planning needs to consider many factors that are not well defined to remodel the foot accordingly, relying on educated decisions from foot surgeons that are based on empirical evidence and experience. Patient outcomes can be varied [16,17,18,19], and therefore, there is some disagreement about optimal surgery for individual patients undergoing this procedure.

This study aims to develop parametric bone models to improve our understanding of the population variance of the calcaneus and medial cuneiform, which are the two bones that are typically reshaped to achieve surgical correction in adolescents with FFF-STA. The most common surgical approach is the Evans and Cotton osteotomies [20] to convert a symptomatic FFF-STA to an asymptomatic FFF. This approach requires a reshaping of the calcaneus and medial cuneiform and lengthening of the calf muscles to release the gastrocnemius fascia [21]. A lateral incision is made over the calcaneus to perform the lateral column lengthening osteotomy (Evans osteotomy) using a tri-cortical bone graft harvested from the iliac crest of the patient [20]. Another incision is made over the medial cuneiform to perform an opening wedge medial cuneiform osteotomy (Cotton osteotomy) [22,23].

There are no studies published that have looked at the variation in the anatomical shape of these two specific bones, the calcaneus and the medial cuneiform, in skeletally mature adolescents with and without flatfoot deformity. Bone variation may be an important factor in the outcomes of flatfoot corrective surgery, and therefore, a study that can quantify and analyze this variation is an essential step in understanding its potential role in patient outcomes. The present paper outlines the methodology to achieve these goals within the context of this study. Principal Component Analysis (PCA) is a dimensionality reduction method commonly used in the medical imaging community to simplify the representation of shape variation. This approach will be applied to a dataset of calcanei and medial cuneiform bones to help improve understanding of variation in the bone morphology of these two bones across the population. The authors note that normal foot and flexible flatfoot demonstrate similar bone morphologies, where the key differences in the flatfoot condition lie in bone positioning under weight-bearing, soft tissue structures and pain experienced by the patient. Thus, using general population data will include both groups and will be analogous to analyzing a sub-set of flexible flatfeet.

## 2. Materials and Methods

The methodology applied in this study is based on the parametrization paper and MATLAB (v. 17, The MathWorks, Inc., Natick, MA, USA) script written by Pascoletti et al. (2021) [24] which was used to analyze the morphological differences in the mandible and the proximal femur. The same general methodology is applied here on the calcaneus and the medial cuneiform of the foot.

### 2.1. Dataset

Initially, five computed tomography (CT) scans of feet from three subjects were used for a preliminary analysis [25]. These were acquired from cadavers, assuming that they did not suffer from any foot-related condition and without specific regard for sex, age or weight. To expand the sample size, the New Mexico Decedent Image Database [26] was used to collect the lower limb CT scans of 10 men and 10 women (40 calcanei and 40 medial cuneiform bones from right and left feet) that have been selected from a database of more than 15,000 subjects who died between 2010 and 2017. The dataset included relevant medical history such as cause of death, any chronic conditions, sex, age and weight. The subjects have been selected by looking at their medical history and by making sure that they did not have any previous pathologies or surgeries that could affect their foot bones’ shapes and that the cause of death did not influence the condition of the foot bones. After further scrutiny, the CT scans of three subjects were not included in the analysis due to prior lower limb surgeries which could have affected the bone morphology of the foot bones and were replaced by three other subjects without foot- and ankle-related issues. Table 1 outlines the subjects that were finally analyzed, which totaled 23 subjects and 45 CT scans of the calcanei and medial cuneiform bones. The sample size used in this study is comparable to the number of bones analyzed in similar bone parametrization studies [24,27,28].

### 2.2. Preparation of the Surface Meshes

**Figure 1:**
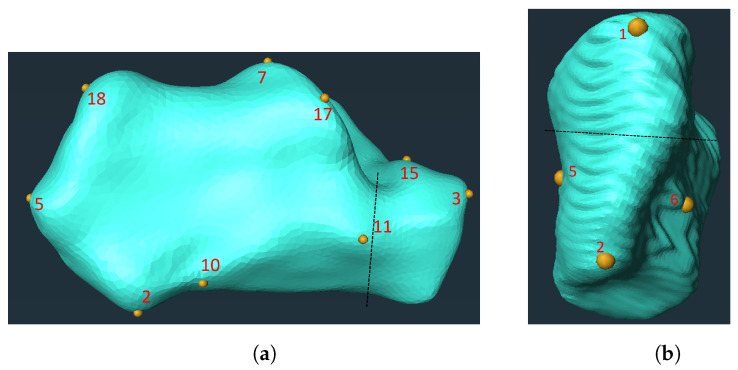
Landmark placement on the calcaneus (**a**) and medial cuneiform (**b**). The dashed black line shows the osteotomy sites.

The CT-scanned images were segmented using the open source software 3D Slicer (v. 5.0.3) with a threshold range of 250 HU (grayscale Hounsfield units) to maximum to create a surface mesh. The surface mesh was re-meshed using an isotropic explicit re-meshing tool in MeshLab (v. 2021.10, open source) to make a smoother homogeneous mesh. Amira (v. 2021.1, Thermo Fisher Scientific, Waltham, MA, USA) was used to place 18 corresponding landmarks on the calcanei and 6 corresponding landmarks on the medial cuneiform bones. Therefore, each bone mesh had the same landmark identification number located on the same anatomical position. The number of landmarks and their locations were defined by the co-author (BB), who is a specialist foot and ankle surgeon (Figure 1, Table 2 and Table 3).

### 2.3. Creation of Iso-Topological Meshes

Transformations were carried out on each individual shape to scale, rotate and locate the models to one individual bone, which was used as a benchmark, so that they were considered as shapes ’only’, which is defined by D.G. Kendall [29] as follows:

…all the geometrical information that remains when location, scale and rotational effects are filtered out from an object.

In this way, all shapes were comparable, and information related to their location, scale and rotation was eliminated. Generalized Procrustes Analysis (GPA) was applied, using the same procedure implemented by Pascoletti [24], to the current dataset in order to find the average shape [30].

In order to perform Principal Component Analysis (PCA) on shapes, it is necessary to have corresponding landmarks in every shape in the training database, to simplify the correspondence problem related to 3D shapes. Iso-topological meshes were created implementing a mesh morphing technique, making the PCA results extensive, where the (x, y, z) coordinates of all nodes of the surface meshes were considered as variables for the PCA (5310 nodes for the calcaneus and 1886 nodes for the medial cuneiform). Hence, all the surface meshes are required to have the same number of nodes, which are connected in the same way and where nodes are identically listed by position order. This resulted in all shapes in the set having identical meshes, with the coordinates of the nodes being the only difference between meshes, based on shape differences.

This can be achieved by many approaches, but here the Radial Basis Function (RBF) method was applied, which consisted of selecting one shape as a ’target mesh’ and using every other shape in the dataset as a ’standard mesh’ that morphed onto the ’target mesh’. The MATLAB script written by Pascoletti [24] ran as follows:

1. The ‘standard mesh’—the mesh that will be morphed—was superimposed onto the ‘target mesh’, eliminating the location and rotational effects, by applying some transformations of rotation and translation, calculated from the landmarks.
2. The mesh was then morphed through the RBF method, and to reproduce a similar shape, all nodes in the standard mesh looked for the closest center of gravity of a triangle in the target mesh and were then projected perpendicularly.
3. A Laplacian smoothing was applied to the standard mesh to improve mesh quality, and then step number 2 was repeated. These two steps were then applied iteratively until the standard mesh fit the target mesh well, without seeing any big gaps that made the surface mesh look irregular.

Having all the shapes in the database aligned, an average shape was calculated using the Procrustes mean (also called the Frechét mean), which is found using Equation (1) [31]:

(1) $$\bar{x}=\frac{1}{N}\sum_{i=1}^{N}x_{i}$$

### 2.4. Principal Component Analysis (PCA)

With 45 iso-topological meshes of the calcanei and medial cuneiform bones completed, the PCA was applied to each database. This led to eigenvectors ($\phi_{i}$) that were the principal components (also called modes) explaining the shape variation and to corresponding eigenvalues ($\lambda_{i}$) that were scalars corresponding to the importance (quantified by the percentage of the variance in the data explained) of the corresponding principal components. Eigenvectors were sorted starting with those with larger eigenvalues, which explain a larger percentage of shape variation.

Using the MATLAB script written by Pascoletti et al. [24,27], the number of principal components needed to be able to describe 90% of the shape variation of the database was found. Once the principal components were calculated and it was decided how many were needed, the average shape of the bone was then compared with the shape described by each PC, considering the principal components one by one in a way that a qualitative analysis could be made, linking a shape variation to each principal component. For example, in order to observe what variation PC1 described, Equation (2) was applied, where $\bar{x}$ was the average shape, $\phi_{1}$ was the mode and $\lambda_{1}$ was the corresponding weight of the first PC.

(2) $${\mathrm{PC}}_{1}=\bar{x}+\phi_{1}\lambda_{1}$$

The script also provided a printout of the coordinates of the landmarks of the analyzed bones once they were all aligned in the same coordinate system. This step was important because the landmarks used were picked by the foot and ankle expert as anatomical landmarks that corresponded to exact points in the bones, so knowing their position allowed for the assessment of how those relevant points varied across the population. These were analyzed using MiniTab (v. 2021, MiniTab, LLC, State College, PA, USA), now applying PCA only on the landmarks’ coordinates.

## 3. Results

**Figure 2:**
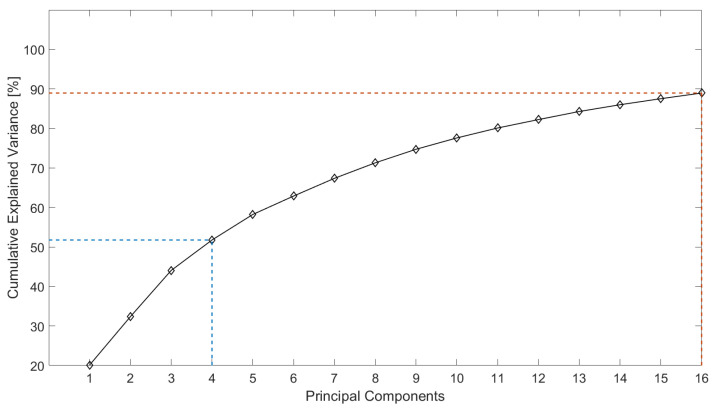
Cumulative explained variance against the number of principal components for the calcaneus, where 16 PCs describe 90% of the shape variation.

**Figure 3:**
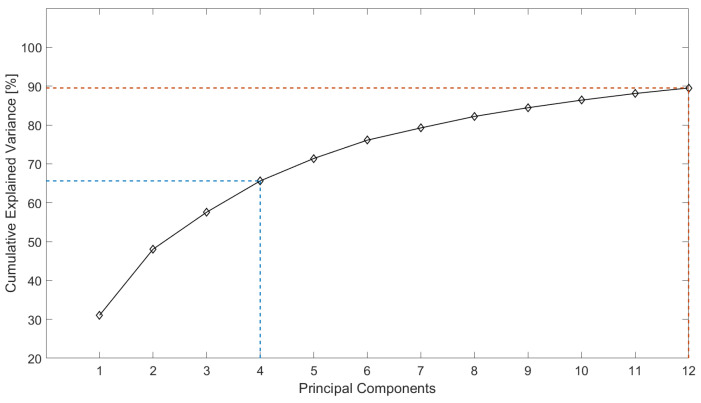
Cumulative explained variance against the number of principal components for the medial cuneiform, where 12 PCs describe 90% shape variation.

Figure 2 and Figure 3 show two graphs of cumulative explained variance for the calcaneus and medial cuneiform datasets, respectively, against the number of principal components that are needed to explain said variance, produced by applying Principal Component Analysis (PCA) on all nodes of the surface meshes of the two datasets. To explain 90% of the total variance of the calcaneus dataset, 16 modes are sufficient, while for the medial cuneiform, 90% of the cumulative variance was explained by 12 modes, as shown in Table 4 and Table 5.

**Figure 4:**
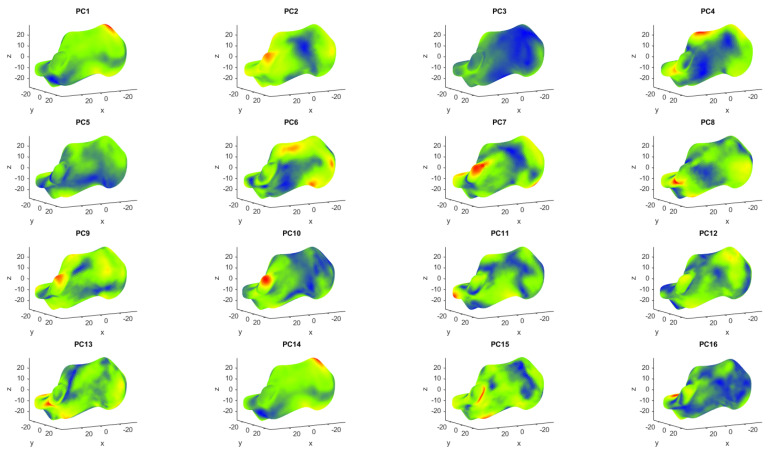
The first 16 PCs, comparing the average calcaneus shape with the respective PC shape, with red areas showing the highest differences and blue areas showing the lowest differences.

**Figure 5:**
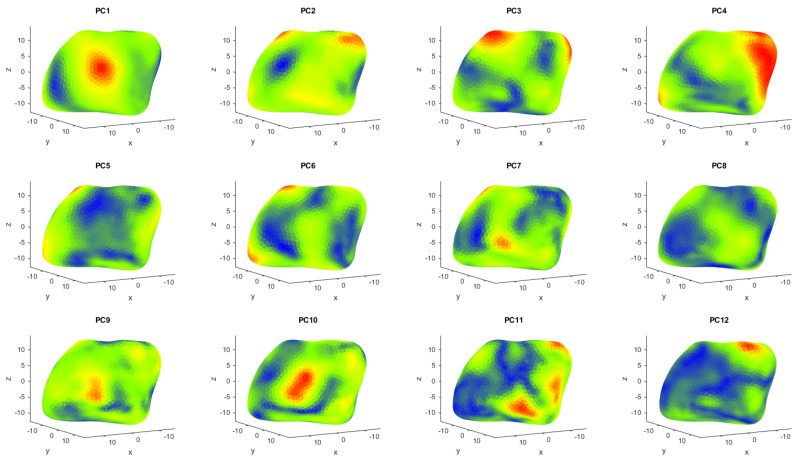
The first 12 PCs, comparing the average medial cuneiform shape with the respective PC, with red areas showing the highest differences and blue areas showing the lowest differences.

**Figure 6:**
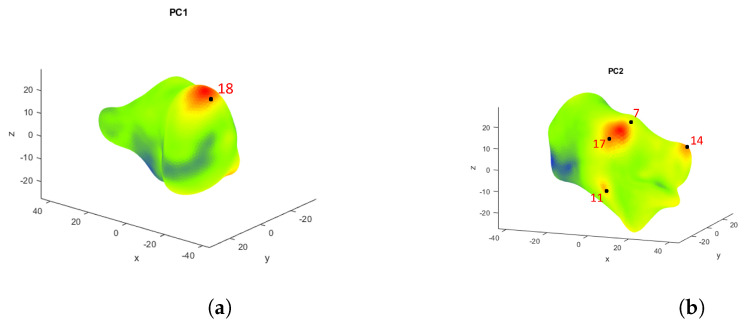
PC1 (**a**) and PC2 (**b**) of the calcaneus dataset, showing in a clearer way the areas highlighted in red, which are the Achilles attachment point (around landmark 18) for PC1 and mainly the posterior talar articular surface (between landmarks 17 and 7), and with the least difference, the fibular trochlea (around landmark 11) and the sustentaculum tali (around landmark 14) for PC2.

The first PC is always the most important eigenvector, explaining the highest variability, with the following PCs describing increasingly less variability within the dataset. For the calcaneus dataset, the first two PCs explain 16.9% and 12.3%, respectively, while for the medial cuneiform, they describe 35.2% and 17.1% of the variance, respectively. All the principal components are represented in Figure 4 and Figure 5 for the two bones, respectively, from the first one in the top left corner, to the last one in the bottom right corner. Each is shown with a colormap where the PC shape is superimposed by the average shape of the bone and the locations showing substantial deviation are highlighted in red. This qualitative analysis of the results of the PCA was made to better understand what each principal component expresses dimensionally. In the colormap of the first PC of the calcaneus dataset (Figure 6), the area with most difference is at the top of the posterior surface of the bone, where the Achilles attachment is located (around landmark 18). Similarly, in the colormap of the second PC, the area with the most difference is the posterior talar articular surface (between landmarks 17 and 7), and then with the least difference there is the fibular trochlea (around landmark 11) and the sustentaculum tali (around landmark 14) [32,33], which are indicated in more detail by the red and yellow areas in Figure 6.

In the colormap of the first PC for the medial cuneiform (Figure 5), the area highlighted in red is in the medial part of the bone (around landmark 5), while in the colormap for the second PC, the areas in red are placed in the superior part of the bone (around landmarks 1 and 2) and in the inferior part close to the neighboring navicular bone (around landmark 4).

**Figure 7:**
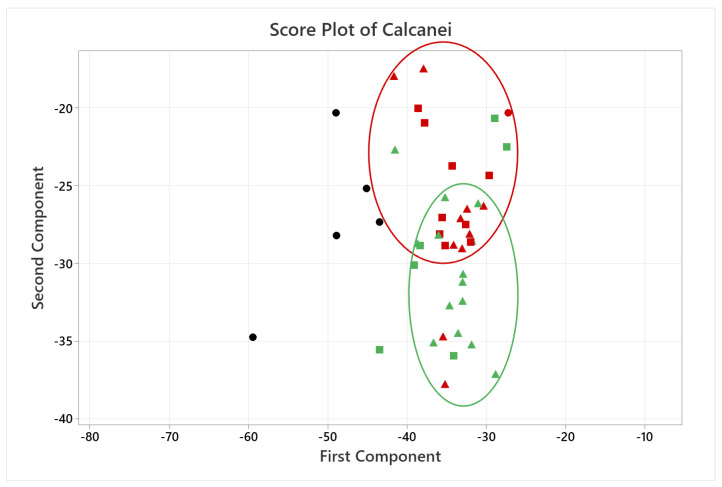
Score plot of the first two PCs for the calcaneus. Points are divided between male (green) and female (red) and healthy BMI (triangles) and high BMI (squares). Black datapoints are the first five CT scans, with no information about the patient.

**Figure 8:**
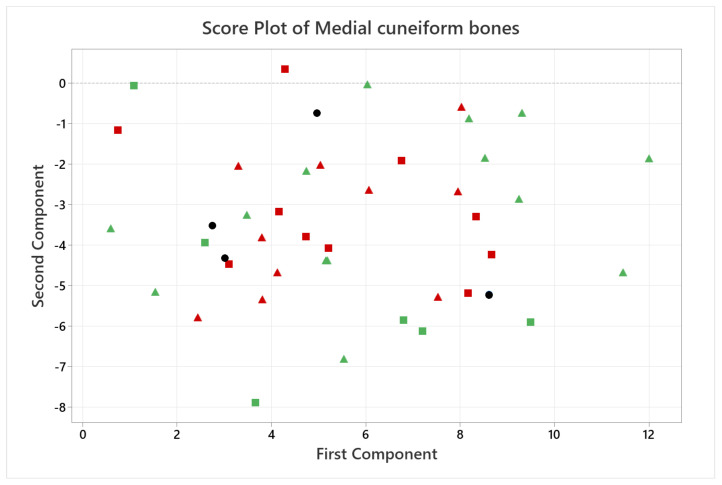
Score plot of the first two PCs for the medial cuneiform. Points are divided between male (green) and female (red) and healthy BMI (triangles) and high BMI (squares). Black datapoints are the first five CT scans, with no information about the patient.

A quantitative analysis was also carried out, applying PCA only on the coordinates of the landmarks, where each landmark became three variables in the analysis, given by its x, y and z coordinates (54 variables for the calcaneus and 18 for the medial cuneiform). Figure 7 and Figure 8 show the score plots of each subject for the calcaneus and medial cuneiform, respectively. For the calcaneus dataset, there is a clear outlier, which is the black point in the lower left corner, which represents one of the first five CT scans with no additional data. It also shows that the female datapoints are clustered together, showing different variance in PC2 compared to male datapoints, also clustered together. For the medial cuneiform dataset, there were no clear outliers and the datapoints of the two sexes are scattered through the plot, showing no evident clusters or trends according to sex or body mass index (BMI) score.

**Figure 9:**
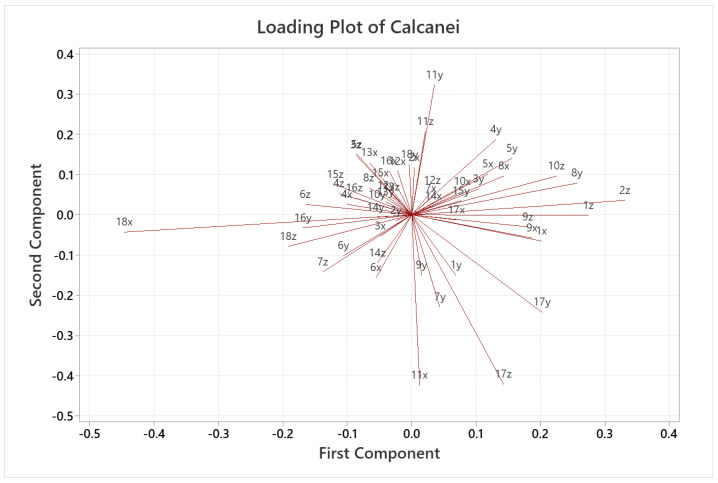
Loading plot for the calcaneus dataset. Variables with longer arrows greatly influence the PC (horizontally PC1 and vertically PC2), and if two arrows are perpendicular, they are completely uncorrelated.

**Figure 10:**
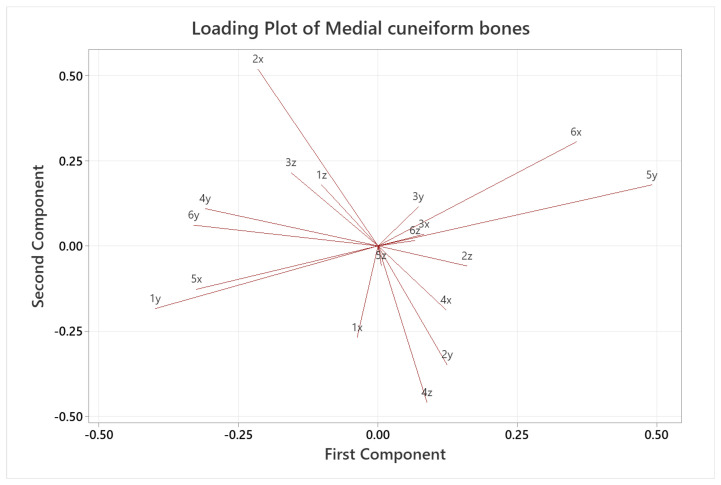
Loading plot for the medial cuneiform dataset. Variables with longer arrows greatly influence the PC (horizontally PC1 and vertically PC2), and if two arrows are perpendicular, they are completely uncorrelated.

Figure 9 and Figure 10 show the loading plots for the two datasets, respectively, where the variables with the largest effect on the first two components are shown according to the length of the lines (magnitude). These are essentially a graphical summary of the correlation structure of the data. Numbers denote the landmark point, and letters denote the Cartesian coordinate (x, y, z) of that landmark. For the calcaneus, point 18x (landmark 18 in the x direction) has a large negative loading on the first component, indicating that the variation in 18x contributed the most to the 20.1% variation in the dataset. Similarly, other landmarks that had large effects include 11y and 11z with large positive loadings and 11x, 17z and 17y with negative loadings on the second component, so these variables contribute the most to the 12.3% shape variation. For the medial cuneiform, the loading plot shows that 5y and 6x have large positive loadings and 1y, 4y, 5x and 6y have negative loadings on the first component (accounting for much of the 31.0% variance), while 2x has a large positive loading and 1x, 2y, 4x and 4z have large negative loadings on the second component, contributing to the 17.0% variability in the shape. A large positive or negative loading (magnitude and direction) gives information on how a variable correlates with another, subject to the caveat this is always approximate, given by the percentage variance explained by a given PC. For example, two arrows going in the same direction are strongly positively correlated between each other (both positives and negatives), meaning that if one variable increases, the other increases, as well. For example, in Table 6, 11y and 11z have almost identical angles to the x-axis (83.8° and 84.2°, respectively); therefore, these two variables are positively correlated. However, two arrows going in opposite directions (almost 180° between them) are strongly negatively correlated, meaning that if one increases, the other decreases. For example, in Table 6, points 2z and 18x are almost completely opposite in direction, meaning that they are negatively correlated. If two arrows are perpendicular relative to each other, they are uncorrelated, such as points 1y and 4z in Table 7. The magnitude and direction of the arrow explain the importance of that variable to either the first or second component. For example, 18x (Table 6) is the most influential landmark for the first component of the calcaneus because of its great magnitude and its angle being almost horizontal on the axis. In comparison, 11x (Table 6) is the most influential landmark for the second component, as shown in the plot, based on the vertical position and magnitude.

## 4. Discussion

Flatfoot surgery is complex and has many variable factors that influence surgical outcomes. Therefore, this study investigates the extent of shape variation in the population as one of these key factors to be understood. This research study examines the bone morphology differences in the calcaneus and medial cuneiform, two bones that are re-shaped during flatfoot surgery to convert a painful flexible flatfoot with short Achilles tendon (FFF-STA) to an asymptomatic flexible flatfoot (FFF). With the help of 18 landmarks for the calcanei and 6 landmarks for the medial cuneiform bones in 45 foot scans, the meshes were altered to be iso-topological meshes, all with the same rotation, location and size. The graphs from the Principal Component Analysis (PCA) show the cumulative explained variance against the principal components. Ninety percent of the total variability can be explained with 16 PCs and 12 PCs for the calcaneus and medial cuneiform datasets, respectively (Figure 2 and Figure 3). This can be explained by the geometry of the bone itself. The calcaneus is the largest bone of the foot and has a complex shape that allows it to transmit the weight of the body to the back of the foot (heel) and to the front of the foot through the cuboid bone [34]. Depending on the forces exerted on the bone, it will change its shape and composition, increasing or lowering its amount of cortical and cancellous bone. The medial cuneiform is a wedge-shaped bone located in the midfoot, acting like the keystone of the medial longitudinal arch of the foot. It has a more regular shape and it is smaller compared to the calcaneus. For these reasons, it seems reasonable to determine that four additional PCs are needed to explain a bigger and hence more complex bone [35] for the calcaneus.

The qualitative colormaps (Figure 4 and Figure 5) and the quantitative loading plots (Figure 9 and Figure 10) present the same data in a way that effectively allows us to observe the anatomical location (colormaps) and quantify the direction and deviation (loading plots) of key shape variations. For example, in the colormap of PC1 for the calcaneus, the area highlighted in red is identified on the loading plot as landmark 18 in the x direction. The same happens for PC2. The location of the osteotomy in the calcaneus is lateral, and although PC2 also describes the area around the fibular trochlea, which is affected by the Evans osteotomy, PC2 mainly explains the area of the posterior talar articular surface, and overall, it describes only 12.3% of the total variance of the whole calcaneus database. The following PCs explain even less than 12.3% each, which is not a considerable contribution to the shape variation. For the medial cuneiform, the most notable observations in Figure 5 are the areas highlighted in red in the colormap for PC1 and PC2, which are confirmed by the loading plot (Figure 10). The location of the Cottons osteotomy is in the superior part of the medial cuneiform, and although PC2 describes only the vertices of the superior part of the bone, it is possible that if we had placed a landmark in the middle of these two points, it would have given us more information about this location and hence inform us about the population variability of this osteotomy site.

This research study informed us that there is not as considerable a difference in bone shape in the calcaneus and medial cuneiform as we thought there would be. PC2 for both datasets could potentially describe the osteotomy sites for both bones. Further studies focusing only on the osteotomy sites in both bones would possibly reveal more information regarding their implications in the surgical planning and outcomes. Another potential study with this dataset would be to investigate how many landmarks are sufficient to identify the shape of the calcaneus, particularly using landmarks that are only visible from the sinus-tarsi approach. The ability to characterize the shape and orientation of a bone from a small surgical cut would potentially allow the possibility of using robotic-guided surgery in the foot and ankle.

Learning about shape patterns on bones could have implications in surgery, as the surgeon will have a better understanding of where shape variation is most critical, and therefore they may influence where a surgeon may cut the bone. For example, this could help planned surgeries to avoid cuts in areas of high shape variation, which could reduce post-surgery complications and uncertainty and improve surgical reliability.

What is most interesting about these shape patterns in the loading plots is that the direction and magnitude of each landmark variable give information about the interdependence between landmarks. This could show coupled or clustered shape changes where one landmark proportionately or inversely proportionally changes another. We have noted from the results that the osteotomy site does not appear to show high variability in the population; this may be an advantage for surgeons as the surgical cuts can be more consistently planned, knowing which bone landmarks showing low population variability could be used as reference points. A research study that focuses on this simplification of the dataset could be useful to reduce the number of landmarks needed to fully identify a bone shape. Due to the complexity of the foot bone shapes, multiple bones and joints in the foot, there are still logistical challenges in implementing robotic surgery in the foot and ankle. However, given the complexity in these surgeries and the many factors affecting surgical outcomes, this study may be a step towards quantifying and understanding bone shape variabilities of the foot. This work could lead to further studies characterizing interdependent anatomical patterns of foot bones, which could be a game-changer for surgical robotics in the foot and ankle to improve outcomes and surgical consistency.

While the score plot for the medial cuneiform (Figure 8) dataset gives no additional information, the score plot for the calcaneus (Figure 7) shows female subjects and male subjects each clustered together, indicating more similarities among them. An anthropology study could further this finding by studying the bone morphology differences of the calcaneus with a gender study and how this could affect the osteotomy location for lateral column lengthening osteotomy or similar foot surgeries.

An important limitation that needs to be mentioned is that the foot and ankle specialist did not position each landmark on the bones meshes himself. The positions of these landmarks were taught by the surgeon (BB) and applied by the main author (YC). Although this means the interpretation of where the landmarks were was decided by a non-clinical researcher, the surgeon identified the number of landmarks and where they should be marked on 2D images of the bones with clinical reasoning as to why he was placing a landmark in that position. This was then implemented by a single person (YC), eliminating any inter-observer variation. Furthermore, analyzing a dataset of only 45 bones to represent a population is another limitation of this research paper. Although Pascoletti et al. [24,27] used 40 and 50 bones, respectively, in their parametrization of the mandible, this is not an extensive dataset in order to reach confident results to be applicable in a surgery setting. Another study that analyzes a bigger pool of subjects’ bones may achieve results that strengthen the results presented here. In order to perform a successful flatfoot surgery, the surgeon needs to consider other patient variabilities that go beyond the shape differences of the calcaneus and medial cuneiform only and would need to include a dataset of clinically diagnosed painful flexible flatfoot with short Achilles tendon (FFF-STA). Despite these limitations, this project was focusing on the bones that are directly cut and reshaped in flatfoot surgery, and therefore, those two bones’ shapes are the most critical variables to look at as a starting point. A further study could look at specific surgical approaches and how critical the position and cutting approach is, considering bone shape variability. Understanding where bone variability is found greatest and least will help with surgical planning in the future.

## 5. Conclusions

The aim of this paper was to increase understanding of the bone morphology and shape variations of the calcaneus and medial cuneiform. This was in order to find an empirical way to assess and plan the reshaping of these two bones in flatfoot surgery, where a surgeon needs to carry out the lateral column lengthening osteotomy on the calcaneus and the opening wedge osteotomy on the medial cuneiform. Using the methodology developed by Pascoletti [24], we were able to understand how these two bones change among a varied population of 45 bones. The analyses show that first principal components (PCs) do not seem to influence the osteotomy sites, but perhaps PC2 of the calcaneus dataset accounts for variation in the lateral part of the bone, which is where the osteotomy site is, and PC2 of the medial cuneiform dataset possibly describes the opening wedge osteotomy site, but further studies examining only the areas around the osteotomy sites should be conducted in order to achieve reliable results that confirm this. The results achieved in this research study show the potential of using statistical shape modeling in this area of flatfoot surgery, proving how further studies analyzing various aspects of the surgery and the population could potentially help and advance this surgery and other types of foot and ankle surgery.

## Figures and Tables

**Table 1:** Table reporting sex, age and body mass index (BMI) of the subjects.

Subject	Sex	Age	BMI (kg/m^2^)
1	Male	19	28.9
2	Male	18	19.8
3	Male	20	21.8
4	Male	18	24.2
5	Male	19	19.0
6	Male	30	26.8
7	Male	49	20.6
8	Male	24	23.3
9	Male	60	31.2
10	Male	20	22.2
11	Female	17	27.3
12	Female	18	33.4
13	Female	18	22.0
14	Female	17	38.4
15	Female	20	23.9
16	Female	64	24.8
17	Female	74	24.9
18	Female	36	60.1
19	Female	60	33.5
20	Female	65	24.8
21	N.A.	N.A.	N.A.
22	N.A.	N.A.	N.A.
23	N.A.	N.A.	N.A.

**Table 2:** Landmark positions on the calcaneus.

# Landmark	Position
#1, #9	Medial process of tuberosity
#2, #10	Lateral process of tuberosity
#12	Anterior tubercle
#11	Fibular trochlea
#14	Sustentaculum tali
#7, #17	Along the border of the posterior articular surface of the talus
#15, #16	Between the articular surface of the cuboid bone and the posterior talar articular surface
#13	Between the anterior and middle articular surface of the talus
#4	Anterior articular surface of the talus
#5, #6	Posterior surface of the calcaneus
#3, #8	Articular cartilage of the calcaneus with the cuboid
#18	Achilles attachment point

**Table 3:** Landmark positions on the medial cuneiform.

# Landmark	Position
#1	Superior vertex (border with the first metatarsal)
#2	Superior vertex (border with the navicular)
#3	Inferior vertex (border with the first metatarsal)
#4	Inferior vertex (border with the navicular)
#5	Middle of the medial aspect
#6	Middle of the lateral aspect

**Table 4:** Table showing the first five principal components for the calcaneus and the variability they explain.

Principal Component	Cumulative Explained Variance (%)	Explained Variance (%)
PC1	20.1	20.1
PC2	32.4	12.3
PC3	44.0	11.7
PC4	51.8	7.7
PC5	58.2	6.5

**Table 5:** Table showing the first five principal components for the medial cuneiform and the variability they explain.

Principal Component	Cumulative Explained Variance (%)	Explained Variance (%)
PC1	31.0	31.0
PC2	48.1	17.0
PC3	57.6	9.5
PC4	65.6	8.1
PC5	71.4	5.8

**Table 6:** Numerical table showing the magnitude and angle of relevant landmarks’ arrows with the positive x-axis for the calcaneus dataset.

Landmark	Magnitude	Angle (°)
2z	0.33	6.2
11x	0.42	−88.4
11y	0.33	83.8
11z	0.21	84.2
17y	0.32	−50.2
17z	0.31	−71.2
18x	0.45	−174.5

**Table 7:** Numerical table showing the magnitude and angle of relevant landmarks’ arrows with the positive x-axis for the medial cuneiform dataset.

Landmark	Magnitude	Angle (°)
1y	0.48	−146.0
2x	0.56	112.5
4z	0.47	−79.2
5y	0.52	20.0

## Data Availability

Data for this manuscript when the article is in print will be available through the Cranfield University CORD data depository and preservation system at https://cranfield.figshare.com (accessed on 22 October 2023), or through the corresponding author s.junaid@aston.ac.uk.

## References

[1] Myerson M.S., Kadakia A.R., Myerson M.S., Kadakia A.R. (2019). Correction of flatfoot deformity in the child. Reconstructive Foot and Ankle Surgery: Management of Complications.

[2] Mosca V.S. (1995). Calcaneal lengthening for valgus deformity of the hindfoot. J. Bone Joint Surg. Am..

[3] Morley A.J.M. (1957). Knock-knee in children. BMJ-Brit Med. J..

[4] Staheli L.T., Chew D.E., Corbett M. (1987). The longitudinal arch. J. Bone Joint Surg. Am..

[5] Pfeiffer M., Kotz R., Ledl T., Hauser G., Sluga M. (2006). Prevalence of flat foot in preschool-aged children. Pediatrics.

[6] Tareco J.M., Miller N.H., MacWilliams B.A., Michelson J.D. (1999). Defining flatfoot. Foot Ankle Int..

[7] Stewart S.F. (1970). Human gait and the human foot: An ethnological study of flatfoot. J. Clin. Orthop. Relat. Res..

[8] Vanderwilde R., Staheli L.T., Chew D.E., Malagon V. (1988). Measurements on radiographs of the foot in normal infants and children. J. Bone Jt. Surg. Am..

[9] Mosca V.S. (2010). Flexible flatfoot in children and adolescents. J. Child Orthop..

[10] Harris R.I., Beath T. (1947). Army Foot Survey.

[11] Carr J.B., Yang S., Lather L.A. (2016). Pediatric pes planus: A state-of-the-art review. Pediatrics.

[12] Ueki Y., Sakuma E., Wada I. (2019). Pathology and management of flexible flat foot in children. J. Orthop. Sci..

[13] Villadot A. (1992). Surgical treatment of the child’s flatfoot. Clin. Orthop. Rel. Res..

[14] Bauer K., Mosca V.S., Zionts L.E. (2016). What’s new in pediatric flatfoot?. J. Pediatr. Orthop..

[15] Phillips G.E. (1983). A review of elongation of os clacis for flat feet. J. Bone Joint Surg..

[16] Kumar S., Sonanis S.V. (2017). Lateral column lengthening for adolescent idiopathic pes planovalgus deformity—Systematic review. J. Orthop..

[17] Suh D.H., Park J.H., Lee S.H., Kim H.J., Park Y.H., Jang W.Y., Baek J.H., Sung H.J., Choi G.W. (2019). Lateral column lengthening versus subtalar arthroereisis for paediatric flatfeet: A systematic review. Int. Orthop..

[18] Jara M.E. (2017). Evans osteotomy complications. Foot Ankle Clin..

[19] Zeifang F., Breusch S.J., Döderlein L. (2006). Evans calcaneal lengthening procedure for spastic flexible flatfoot in 32 patients (46 feet) with a followup of 3 to 9 years. Foot Ankle Int..

[20] Mosca V.S. (2014). Management of the painful adolescent flatfoot. Tech. Foot Ankle Surg..

[21] Lee K.M., Jung H.G., Jung H.G. (2016). Congenital flatfoot, painful accessory navicular, and tarsal coalition. Foot and Ankle Disorders: An Illustrated Reference.

[22] Tennant J.N., Carmont M., Phisitkul P. (2014). Calcaneus osteotomy. Curr. Rev. Musculoskelet Med..

[23] Vulcano E., Maccario C., Myerson M.S. (2016). How to approach the pediatric flatfoot. World J. Orthop..

[24] Pascoletti G., Aldieri A., Terzini M., Bhattacharya P., Calì M., Zanetti E.M. (2021). Stochastic PCA-based bone models from inverse transform sampling: Proof of concept for mandibles and proximal femurs. Appl. Sci..

[25] Cai Y., Junaid S., Budair B., Pascoletti G., Zanetti E.M., Ringrose T., Zioupos P. Parametrisation of the calcaneus and medial cuneiform. Proceedings of the 28th Congress of the European Society of Biomechanics.

[26] Edgar H.J.H., Daneshvari B.S., Moes E., Adolphi N.L., Bridges P., Nolte K.B. New Mexico Decedent Image Database. Office of the Medical Investigator, University of New Mexico. https://nmdid.unm.edu/.

[27] Pascoletti G. (2022). Statistical shape modelling of the human mandible: 3D shape predictions based on external morphometric features. Int. J. Interact. Des. Manuf..

[28] Pascoletti G., Calì M., Bignardi C., Conti P., Zanetti E.M. Mandible morphing through principal component analysis. Proceedings of the International Conference on Design Tools and Methods in Industrial Engineering.

[29] Dryden I.L., Mardia K.V. (1998). Statistical Shape Analysis.

[30] Lindner C., Zheng G., Li S., Szekely G. (2017). Automated image interpolation using statistical shape models. Statistical Shape and Deformation Analysis.

[31] Stegmann M.B., Gomez D.D. (2002). A Brief Introduction to Statistical Shape Analysis.

[32] Standring S. (2021). Gray’s Anatomy.

[33] Netter F.H. (2023). Netter Atlas of Human Anatomy: Classic Regional Approach.

[34] Krmek V., Krmek N., Jo-Osvatić A., Nikolić V. (2011). Antropological measurement of the calcaneus. Coll. Antropol..

[35] Zhou E., Lui J. (2021). Physiological regulation of bone length and skeletal proportion in mammals. Exp. Physiol..

